# Supplementary figures and images for: Genome-Wide Identification of Glutathione S-Transferase Genes in Eggplant (Solanum melongena L.) Reveals Their Potential Role in Anthocyanin Accumulation on the Fruit Peel
Source: Int J Mol Sci. 2024 Apr 11;25(8):4260. doi: 10.3390/ijms25084260 (PMC11050406; doi:10.3390/ijms25084260)

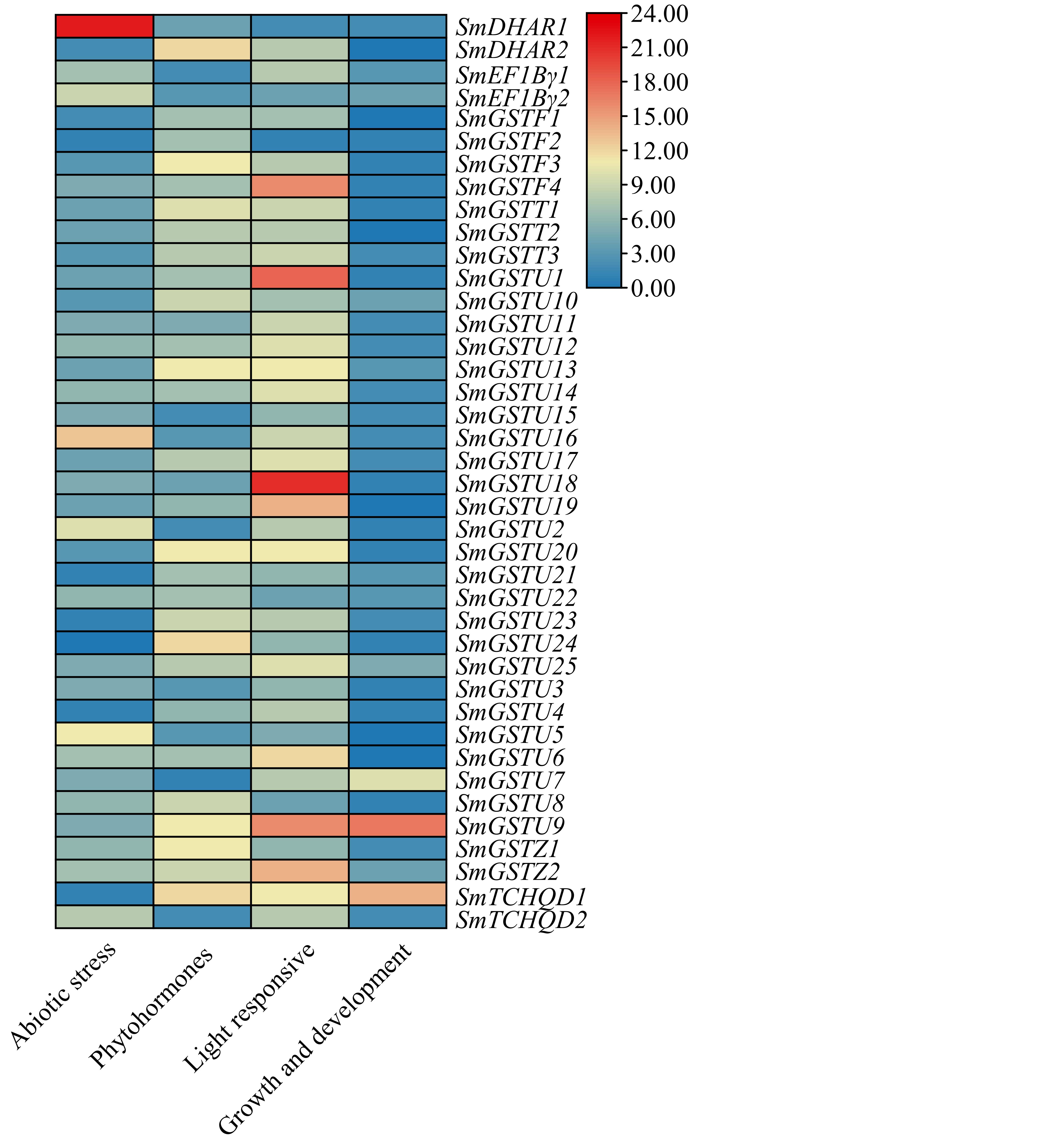

Supplement: Supplementary file 1 [file ijms-25-04260-s001.zip › supplementary file/Figure S1.jpg]
